# Vasculogenic Mimicry Formation Is Associated with Erythropoietin Expression but Not with Erythropoietin Receptor Expression in Cervical Squamous Cell Carcinoma

**DOI:** 10.1155/2019/1934195

**Published:** 2019-02-21

**Authors:** Yue-Jie Li, Xin Qing, Qing-Xu Tao, Li Xiang, Li Gong, Tie-Jun Zhou

**Affiliations:** ^1^Department of Pathology, Affiliated Hospital of Southwest Medical University, Luzhou, Sichuan province, China; ^2^School of Clinical Medicine, Southwest Medical University, Luzhou, Sichuan province, China; ^3^Department of Pathogenic Biology, Southwest Medical University, Luzhou, Sichuan province, China

## Abstract

**Background:**

Vasculogenic mimicry (VM), as an endothelium-independent cancer microcirculation, has been observed in many malignancies including cervical cancer. Erythropoietin (EPO) and erythropoietin receptor (EPO-R) could produce an angiogenic effect to promote cervical squamous cell carcinoma (CSCC) progression. However, the association between VM formation and EPO/EPO-R expression in CSCC is poorly explored.

**Methods:**

Seventy-six paraffin-embedded CSCC samples, 25 high-grade squamous intraepithelial lesion (HSIL) samples, 20 low-grade squamous intraepithelial lesion (LSIL) samples, and 20 normal cervix samples were collected. Immunohistochemistry SP method was performed to detect EPO/EPO-R expression and CD31/periodic acid-Schiff (PAS) double staining was performed to detect VM formation. The associations of EPO/EPO-R and VM with clinicopathological parameters of CSCC were analyzed. The associations between VM formation and EPO/EPO-R expression were also analyzed.

**Results:**

The positive expression rates of EPO and EPO-R were gradually increasing along the progression of normal cervix-LSIL-HSIL-CSCC sequence (*P*<0.05). EPO and EPO-R expression were not significantly associated with clinicopathological parameters of CSCC patients (*P*>0.05). VM was significantly associated with FIGO stage, lymphovascular space involvement, and lymph node metastasis (*P*<0.05). VM was positively associated with EPO expression (*r*=0.284,* P*<0.05) but was not associated with EPO-R expression (*P*>0.05).

**Conclusion:**

These data suggest that increased EPO/EPO-R expression may play an important role in cervical carcinogenesis. EPO overexpression may promote VM formation in CSCC.

## 1. Introduction

Cervical cancer ranked as the fourth most frequently diagnosed cancer and fourth leading cause of cancer death in women. It was estimated that there were 570,000 new cases and 311,000 deaths of cervical cancer worldwide in 2018 [1]. Cervical squamous cell carcinoma (CSCC) represents the most common histologic subtype of cervical cancer and accounts for 85-90% of cases [2]. Cervical carcinogenesis is a very complex multistep progression. Normal cervix first progresses to low-grade squamous intraepithelial lesions (LSIL) and then to high-grade squamous intraepithelial lesions (HSIL), thus eventually shaping CSCC. Although persistent infection with high-risk human papillomavirus (HPV), such as HPV16 and HPV18, is well known as the cause of CSCC and CSSS related precancerous lesions, there exist more pathogenic factors in CSCC progression [2, 3].

Tumor angiogenesis is an essential hallmark of malignancy. Successful cervical carcinogenesis depends on angiogenesis, because intratumoral angiogenesis not only supplies nutrients and oxygen for tumor growth but also provides a pathway for tumor distant dissemination [4]. Noteworthily, recent evidence suggested that endothelium-dependent angiogenesis is not an exclusive pattern of tumor microcirculation. An endothelium-independent tumor blood supply pattern called vasculogenic mimicry (VM) was introduced to describe the phenomenon that highly aggressive cancer cells could form vasculogenic-like networks, comprised of a basement membrane with periodic acid-Schiff (PAS) staining positive and lined cancer cells with CD31 Immunohistochemical staining negative [5, 6]. As an endothelium-independent pathway, VM is closely implicated in cancer malignant degree due to its unique structure. VM channels have connections with host endothelium-dependent microvessels. Cancer cells lining the inner surface of VM channels are directly exposed to blood flow, possess high plasticity, and could secret proteinase to degrade basement membrane or matrix [7, 8]. These features imply that VM, the novel pattern of tumor perfusion, might more easily contribute to cancer early invasion and metastasis [9, 10]. Therefore, it is very necessary to seek promising therapeutic targets for anti-VM in cancer progression. Although several molecules like phosphoinositide 3 kinase (PI3K) and matrix metalloproteinases (MMPs) have been identified to be involved in VM formation [11], the detailed molecular mechanisms underlying VM still need to be further explored.

Erythropoietin (EPO) is a kind of glycoprotein hormone that could regulate erythroid cells proliferation and differentiation by binding to its specific transmembrane erythropoietin receptor (EPO-R), which belongs to the cytokine receptor type I superfamily. It has been long believed that EPO is generally produced by fetal liver or adult kidney and acts only on erythropoiesis [12, 13]. Over the last few decades, increasing studies have revealed that EPO/EPO-R express in many nonhematopoietic cells and tissues and play mitogenic, antiapoptotic, and tissue-protective roles. Noteworthily, EPO/EPO-R expression is also found in various solid tumors where they exert an obvious function of inducing angiogenesis [14, 15]. Serving as the malignant solid tumor of female reproductive tract, CSCC could widely and strongly express EPO/EPO-R to induce tumor angiogenesis, thus promoting cancer progression [16, 17].

To date, conclusion that EPO/EPO-R promotes endothelium-dependent angiogenesis in cancer has been well established. Considering VM is another pattern of tumor microcirculation, there are, however, few report concerning whether EPO/EPO-R promotes tumor VM formation too. Therefore, this study aimed to explore whether VM formation is associated with EPO/EPO-R expression in CSCC and assess their correlations with clinicopathological parameters of CSCC.

## 2. Materials and Methods

### 2.1. Patients and Tissue Samples

A total of 141 paraffin-embedded cervical tissues were obtained from the Affiliated Hospital of Southwest Medical University between January 2010 and December 2011, including 76 CSCC samples, 25 HSIL samples, 20 LSIL samples, and 20 normal cervix samples. Normal cervix samples were obtained from patients undergoing radical hysterectomy for uterine leiomyoma. All pathological diagnoses were reconfirmed by pathologists based on hematoxylin and eosin (HE) staining. All patients enrolled in this study had complete clinical data and none of them received preoperative radiotherapy, chemotherapy, or hormonal therapy. Ages of the 76 CSCC patients ranged from 28 to 62 years, and the mean age of CSCC patients was 44.85±7.01 years old. CSCC cases were staged according to the International Federation of Gynecology and Obstetrics (FIGO) 2000 staging system and the clinicopathological parameters of CSCC patients are summarized in Table 2.

### 2.2. Immunohistochemical (IHC) Staining

All tissue samples were fixed in neutral formalin, embedded in paraffin, cut into 4 *μ*m thick sections, and placed on glass slides. IHC staining was performed by a Biotin-Streptavidin HRP Detection Kit (Zhongshan Golden Bridge Biotechnology Limited Company, Beijing, China). After baking at 60°C overnight, tissue sections were deparaffinized in xylene and rehydrated in graded alcohols. For antigen retrieval, the sections were heated for 8 minutes in EDTA by using a pressure cooker and then cooled naturally to room temperature, followed by immersing the sections into 3% hydrogen peroxide in methanol for 10 minutes to exhaust endogenous peroxidase activity. After washing with phosphate-buffered saline (PBS), nonspecific binding was blocked with normal goat serum for 15 minutes. Next the sections were incubated with rabbit anti-EPO polyclonal antibody (1:100 dilution; Bioworld Technology, USA) and rabbit anti-EPOR polyclonal antibody (1:100 dilution; Beijing Biosynthesis Biotechnology, Beijing, China) overnight at 4°C. After washing the primary antibody off, the sections were incubated with biotinylated secondary antibody and followed by horseradish peroxidase-labeled streptavidin from the kit. Immunoreactivity was visualized by DAB(3,3-diaminobenzidine) and then the sections were counterstained with hematoxylin, dehydrated, dried, and mounted. Known positive and negative controls were also performed. Ultimately the sections were ready for microscopic examination.

Staining results were evaluated independently by two experienced pathologists who were blinded to the clinical data according to both the intensity of staining and the proportion of positively stained cells. The intensity of staining was scored as follows: negative as 0, light yellow as 1, yellow brown as 2, and brown as 3. The proportion of positively stained cells was scored as follows: 0–10% positive tumor cells as 0, 11%–40% positive tumor cells as 1, 41%–70% positive tumor cells as 2, and 71%–100% positive tumor cells as 3. The final scores (range 0–6) were calculated by adding the intensity of staining scores and the proportion of positively stained cells scores, 0–1 final scores were defined as negative expression, and the others were defined as positive expression [18].

### 2.3. CD31/Periodic Acid-Schiff (PAS) Double Staining

IHC staining for CD31 (1:200 dilution; Zhongshan Golden Bridge Biotechnology Limited Company, Beijing, China) was performed as mentioned above. In briefly, after using DAB as the chromogen, the sections were washed with distilled water and incubated with periodic acid solutions for 10 minutes and then Schiff solutions for 30–60 minutes by using a PAS staining kit (Nanjing Jiancheng Biotechnology Limited Company, Nanjing, China). Last the sections were counterstained with hematoxylin, dehydrated, dried, and mounted.

Subsequently, CD31/PAS double staining sections were viewed at magnification 400. CD31 positively stained channels were considered as the endothelium-dependent vessels. Combining corresponding HE staining sections, VM was defined as PAS–positive channels lined by tumor cells rather than CD31 labeled endothelial cells where erythrocytes may be present in the lumen.

### 2.4. Statistical Analysis

Statistical analyses were conducted with SPSS 17.0 software (Chicago, IL, USA). The Chi–square test was used to analyze the associations of EPO, EPO-R, and VM with clinicopathological parameters of CSCC. Spearman's rank test was used to analyze the associations between VM formation and EPO/EPO-R expression. A two-tailed* P* <0.05 was considered statistically significant.

## 3. Results

### 3.1. EPO/EPO-R Expression and VM Formation in Normal Cervix, LSIL, HSIL, and CSCC

**Figure 1:**
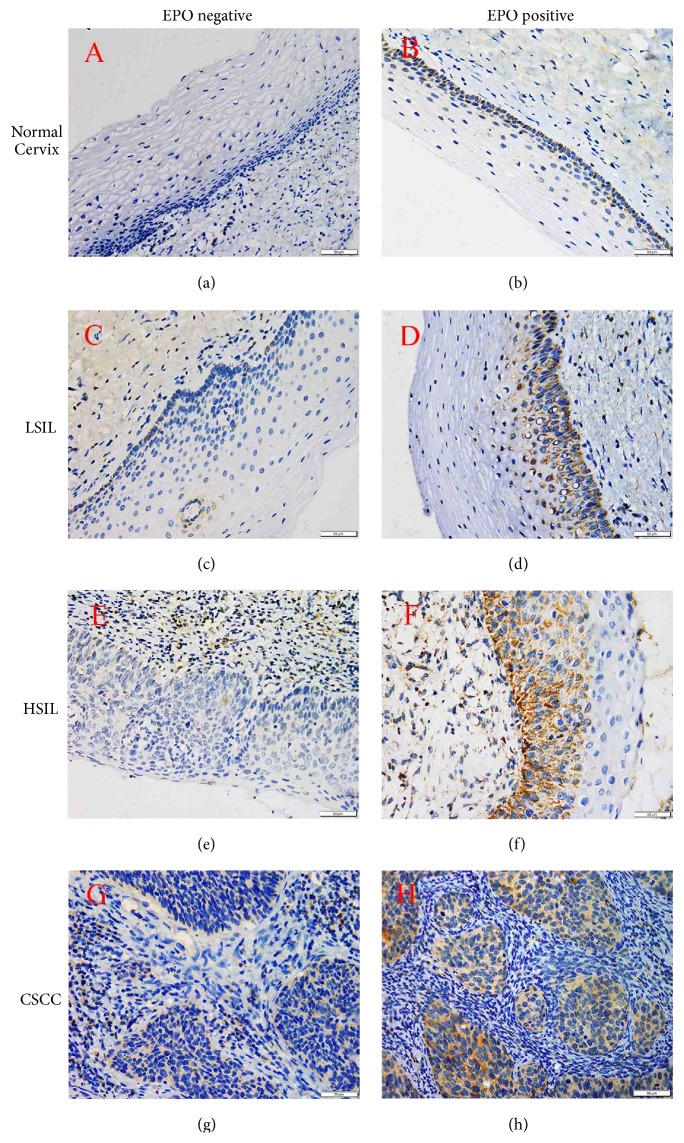
Immunostaining for EPO expression (400×magnification). a, c, e, and g negative expression of EPO in normal cervix, LSIL, HSIL, and CSCC, respectively; (b, d, f, and h) positive expression of EPO in normal cervix, LSIL, HSIL, and CSCC, respectively. As shown above, EPO expression is presented with weak and localized staining in the cytoplasm of basal cells in normal cervix. By contrast, in the tissues of LSIL, HSIL, and CSCC, EPO expression gradually becomes more strong and diffuse in the cytoplasm of cancer cells and atypical cells. The immune-staining intensity significantly increased from normal cervix to LSIL to HSIL and then to CSCC.

**Figure 2:**
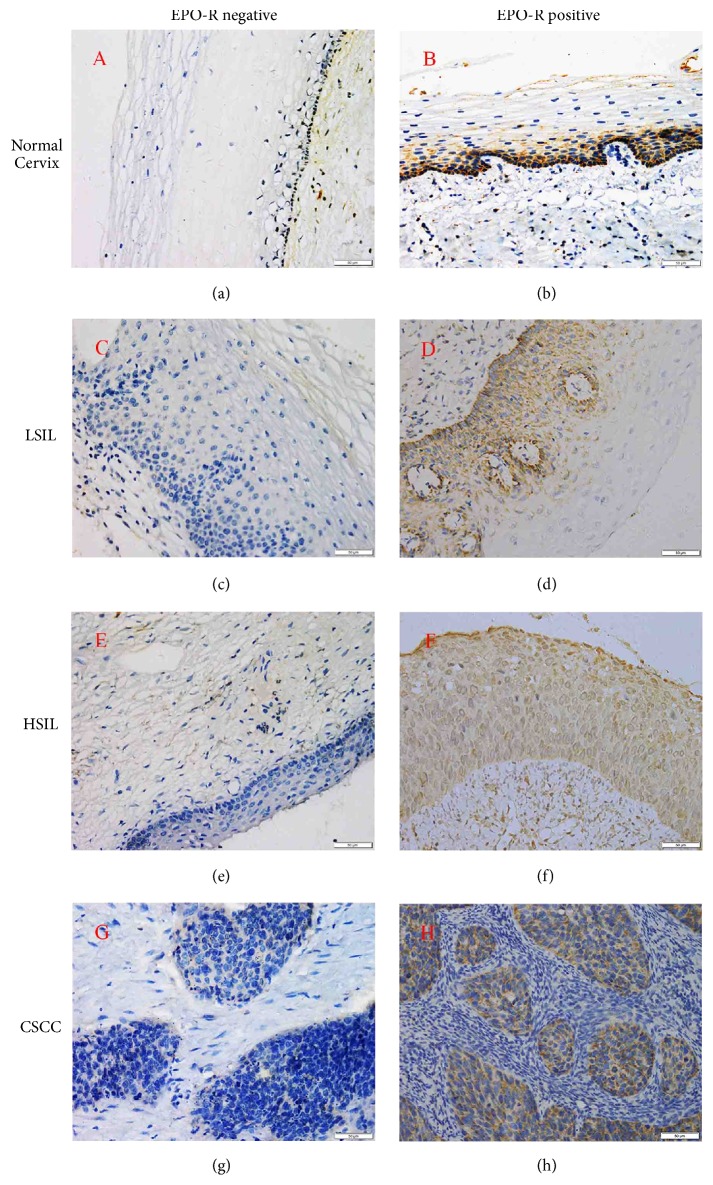
Immunostaining for EPO-R expression (400×magnification). a, c, e, and g negative expression of EPO-R in normal cervix, LSIL, HSIL, and CSCC, respectively; (b, d, f, and h) positive expression of EPO-R in normal cervix, LSIL, HSIL, and CSCC, respectively. As shown above, EPO-R expression is presented with moderate and diffuse staining in the cytoplasm of basal and spinous cells. By contrast, in the tissues of LSIL, HSIL, and CSCC, EPO-R expression is a strong and diffuse staining in the cytoplasm of cancer cells and atypical epithelial cells. A significantly increased immunostaining intensity from normal cervix to LSIL to HSIL and to CSCC could be also observed.

Representative patterns of EPO and EPO-R expression were shown in Figures 1 and 2, respectively. A diffuse and strong cytoplasmic EPO expression was observed in dysplastic cells and cancer cells. Moderate EPO expression was also observed in some normal cervical squamous epithelial and glandular epithelial cells. EPO-R expression was mainly localized in cytoplasm and cytomembrane. In some cases, endothelial cells also expressed EPO-R and nonspecific IHC reactivity of EPO-R was found in this study. The immunostaining intensities of EPO and EPO-R were both significantly increasing from normal cervix to LSIL to HSIL and then to CSCC.

As presented in Table 1, the positive expression rates of EPO in normal cervix, LSIL, HSIL, and CSCC were 10.00% (2/20), 25.00% (5/20), 52.00% (13/25), and 76.32% (58/76), respectively, and the differences had statistical significance (*χ*^2^ =37.724,* P*≤0.001). The positive expression rate of EPO in CSCC was significantly higher than that in normal cervix (76.32% vs. 10.00%,* P*≤0.001) and that in HSIL (76.32% vs. 52.00%,* P*=0.021). The positive expression rates of EPO-R in normal cervix, LSIL, HSIL, and CSCC were 15.00% (3/20), 30.00% (6/20), 60.00% (15/25), and 82.89% (63/76), respectively, and the differences had statistical significance (*χ*^2^ =41.441, P≤0.001). The positive expression rate of EPO-R in CSCC was significantly higher than that in normal cervix (82.89% vs. 15.00%,* P*≤0.001) and that in HSIL (82.89% vs. 60.00%, P=0.018). The positive expression rate of EPO-R between HSIL and LSIL also had statistically significant difference (60.00% vs. 30.00%, P=0.045).

**Figure 3:**
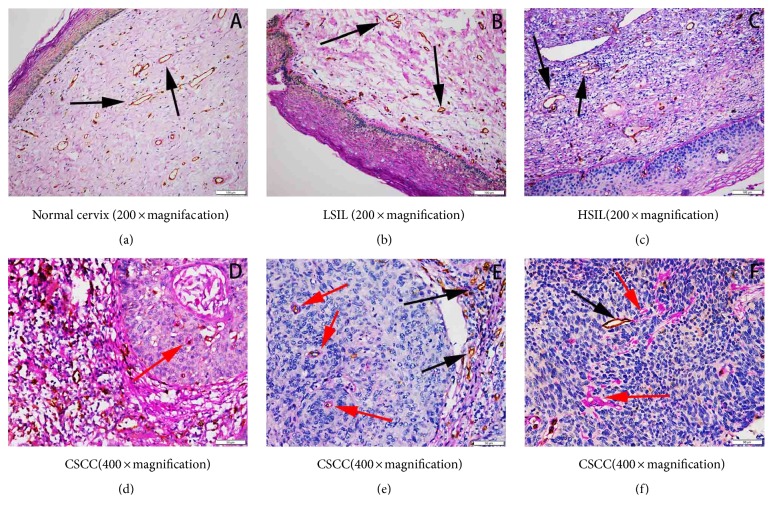
CD31/PAS double staining for showing VM morphology. a, b, and c demonstrated the typical blood vessels in normal cervix, LSIL, and HSIL, respectively (200×magnification). d, e, and f demonstrated VM channels and typical blood vessels in CSCC (400×magnification). Black arrows indicate typical microvessels with CD31 positive and PAS negative. Red arrows indicate VM channels consisting of cancer cells and basement membrane, with CD31 negative and PAS positive correspondingly.

VM formation was only observed in CSCC samples and its positive rate was 35.53% (27/76). Morphological characteristics of VM in CSCC were shown in Figure 3, the VM channels consisted of CSCC cells, and basement membranes were negative for CD31 but positive for PAS staining. Neither infiltrating inflammatory cells nor necrosis was seen around the channels.

### 3.2. The Associations of EPO/EPO-R Expression and VM Formation with Clinicopathological Parameters of CSCC

The associations of EPO/EPO-R expression and VM with clinicopathological parameters of CSCC were presented in Table 2. Both EPO and EPO-R expressions were not significantly associated with any clinicopathological parameters of CSCC patients (P>0.05). VM formation was significantly associated with FIGO stage (*χ*^2^ =8.146, P=0.004), lymphovascular space involvement (*χ*^2^ =12.380, P≤0.001), and lymph node metastasis (*χ*^2^ =11.611, P≤0.001) but not associated with the other clinicopathological parameters of CSCC patients (P>0.05). Further correlation analysis found that VM formation was positively associated with FIGO stage (r=0.327, P=0.004), lymphovascular space involvement (r=0.404, P≤0.001), and lymph node metastasis (r=0.391, P≤0.001).

### 3.3. The Associations of VM Formation with EPO/EPO-R Expression of CSCC

As presented in Table 3, the positive expression rate of EPO in VM positive group was significantly higher than that in VM negative group (*χ*^2^ =6.138, P=0.013). Further correlation analysis found that VM formation was positively associated with EPO expression (r=0.284, P=0.013). VM formation was not associated with EPO-R expression (P>0.05).

## 4. Discussion

Oxygen is an indispensable element for cellular metabolism. But tumors, especially malignant solid tumors, are often susceptible to tissue hypoxia when the tumor mass reaches a volume that restricts sufficient oxygen to diffuse in tumor central regions. Consequently, a series of signaling pathways are activated by tumor cells to adapt to this hypoxic microenvironment and maintain sustaining growth. Hypoxia-inducible factors (HIFs), undoubtedly, play a powerful mediator in the whole process [19, 20]. It is widely accepted that HIF-1*α* could turn on the switch of angiogenesis by cross talking with numerous growth factors, which is a crucial way to relive oxygen starvation of cervical cancer [21, 22]. VM, known as an angiogenesis-independent blood supply pattern, is also tightly regulated by HIF-1*α*. Recent studies indicated that hypoxia promotes VM formation and knockdown of HIF-1*α* expression inhibits VM formation [23, 24]. Similarly, study indicated hypoxia stimulates EPO synthesis and loss of HIF-2 impairs EPO expression [25].

Concretely speaking, the key VM signaling cascade is initiated by HIFs and HIFs can transcriptionally upregulate the expression of vascular endothelial (VE)-cadherin. VE-cadherin induces erythropoietin-producing hepatocellular carcinoma-A2 (EphA2) to interact with its membrane bound ligand and become phosphorylated. Phosphorylated EphA2 subsequently activates PI3K in a direct or focal adhesion kinase (FAK) and extracellular signal-regulated kinase 1/2 (ERK1/2) dependent way. Besides, vascular endothelial growth factor-A (VEGF-A) and VEGF-receptor1 are also shown to participate in this pathway. As a result, MMPs activation and laminin 5*γ*2 cleavage eventually lead to VM formation [26]. Interestingly, abundant studies also proved that EPO could activate PI3K signaling in ischemia reperfusion injury [27, 28]. EPO gene could induce ERK1/2 phosphorylation and increase MMP-9 expression in both bladder cancer cells and vascular smooth muscle cells [29, 30]. Besides, EPO-R could be activated via VEGF-A mediated interaction between EPO-R and VEGF-receptor 2 [31]. Obviously, from what has been discussed above, we can easily find that both VM formation and EPO/EPO-R expression are involved in many common signaling molecules of HIFs, PI3K, ERK1/2, MMPs, and VEGF-A. Therefore relevant questions were naturally brought to us: Does EPO/EPO-R expression influence VM formation through regulation of these intermediate signaling molecules? Is there any association between VM formation and EPO/EPO-R expression?

Therefore, in the present study, we detected EPO/EPO-R expression and VM formation in CSCC samples by using IHC staining. We found a significantly positive association between VM formation and EPO expression, which made us hypothesize that EPO expression may promote VM formation in CSCC. In order to figure out whether there exists causal relationship between EPO expression and VM formation, future studies are required to further investigate the VM networks formation in vitro three-dimensional (3D) culture following the treatment of EPO knockdown and EPO overexpression. We also found that VM formation was significantly associated with FIGO stage, lymphovascular space involvement, and lymph node metastasis of CSCC patients, which agrees with the viewpoint that VM might more easily contribute to cancer invasion and metastasis due to its unique structure [9, 10]. VM serves as an aggravating supplement to tumor microcirculation, and single antiangiogenic therapy tends to be insufficient to cut tumor blood supply. Targeting both angiogenesis and VM formation is becoming an important challenge [7].

In our study, we found that EPO expression was significantly associated with VM formation, indicating that EPO can not only promote tumor angiogenesis but also facilitate VM formation in CSCC. However, in our further analysis, EPO-R failed to be related to VM, indicating that EPO-R may not be involved in VM in CSCC. The negative association of EPO-R with VM entailed us hypothesizing that there might be more EPO related mechanisms beyond EPO-R causing to VM formation. it has been reported that EPO could switch on tumor angiogenesis just by activating JAK2/STAT5 and PI3K/Akt signal pathways [32]. EPO could directly promote vascular endothelial cells proliferation and induce angiogenesis by upregulating the expression of VEGF, VEGFR2, HGF, and FGF-2, which are key contributors in angiogenesis [33]. Therefore, whether these EPO-R-independent pathways would also be responsible for EPO-induced VM formation deserves further investigation.

This study also showed that both the positive expression rates of EPO and EPO-R were gradually increasing along the normal cervix-LSIL-HSIL-CSCC progression, which suggests that increased EPO/EPO-R expression may promote CSCC progression [16]. In addition, EPO is closely related to clinical applications. Anemia is a frequent complication of cancer patients, which is derived from either cancer itself or chemoradiotherapy. Aiming at anemia correction, administering recombinant human EPO (rHuEPO) could improve patients' hemoglobin (Hb) levels and exert substantial clinical benefits [34]. Controversially, later researches also reported that there exist potential risks from rHuEPO administration, such as accelerating tumor proliferation, promoting tumor angiogenesis, thus increasing mortality rates of these patients. For the past few decades, the dilemma of rHuEPO treatment for cancer patients has been a heated discussion. A more comprehensive understanding of rHuEPO treatment is still needed so far [35–38]. If EPO expression is proved to be a promotion to VM formation in further study, this may remind us of caution to VM formation when cancer patients receive rHuEPO administration.

In conclusion, the present study showed evidence that VM formation was positively associated with EPO expression in CSCC by using IHC staining. EPO overexpression may promote VM formation of CSCC. Our findings may provide new insights into the molecular mechanisms underlying VM formation.

## Figures and Tables

**Table 1:** IHC expression of EPO/EPO-R and VM formation in normal cervix, LSIL, HSIL and CSCC, respectively.

Group	n	EPO				EPO-R				VM			
		- (%)	+ (%)	*χ* ^2^	*P*	- (%)	+ (%)	*χ* ^2^	*P*	- (%)	+ (%)	*χ* ^2^	*P*
Normal cervix	20	18(90.00)	2(10.00)	1.558	0.212^a^	17(85.00)	3(15.00)	1.290	0.256^a^	20(100.00)	0(0.00)		
LSIL	20	15(75.00)	5(25.00)	3.375	0.066^b^	14(70.00)	6(30.00)	4.018	0.045^b*∗*^	20(100.00)	0(0.00)		
HSIL	25	12(48.00)	13(52.00)	5.327	0.021^c*∗*^	10(40.00)	15(60.00)	5.607	0.018^c*∗*^	20(100.00)	0(0.00)	9.886	0.002^c*∗*^
CSCC	76	18(23.68)	58(76.32)	29.709	≤0.001^d*∗*^	13(17.11)	63(82.89)	33.972	≤0.001^d*∗*^	49(64.47)	27(35.53)	9.886	0.002^d*∗*^

a. normal cervix versus LSIL; b. LSIL versus HSIL; c. HSIL versus CSCC; d. CSCC versus normal cervix.

*∗* P<0.05, statistically significant

**Table 2:** Associations of EPO/EPO-R expression and VM formation with clinicopathological parameters of CSCC.

Parameter	n	EPO				EPO-R				VM			
		- (%)	+ (%)	*χ* ^2^	*P*	- (%)	+ (%)	*χ* ^2^	*P*	- (%)	+ (%)	*χ* ^2^	*P*
Age(years)													
⩽40	30	5(16.67)	25(83.33)	1.350	0.245	4(13.33)	26(86.67)	0.497	0.481	19(63.33)	11(36.67)	0.028	0.867
>40	46	13(28.26)	33(71.74)			9(19.57)	37(80.43)			30(65.22)	16(34.78)		
Tumor size(cm)													
⩽4	48	11(22.92)	37(77.08)	0.042	0.837	8(16.67)	40(83.33)	0.018	0.894	33(68.75)	15(31.25)	1.040	0.308
>4	28	7(25.00)	21(75.00)			5(17.86)	23(82.14)			16(57.14)	12(42.86)		
FIGO stage													
I	42	11(26.19)	31(73.81)	0.326	0.568	8(19.05)	34(80.95)	0.250	0.617	33(78.57)	9(21.43)	8.146	0.004*∗*
II	34	7(20.59)	27(79.41)			5(14.71)	29(85.29)			16(47.06)	18(52.94)		
Histological types													
Keratinizing	25	8(32.00)	17(68.00)	1.425	0.233	7(38.00)	18(72.00)	3.119	0.077	19(76.00)	6(24.00)	2.161	0.142
Non-keratinizing	51	10(19.61)	41(80.39)			6(11.76)	45(88.24)			30(58.82)	21(41.18)		
Lymphovascular space involvement													
Positive	33	5(15.15)	28(84.85)	2.349	0.125	3(9.09)	30(90.91)	2.642	0.104	14(42.42)	19(57.58)	12.380	≤0.001*∗*
Negative	43	13(30.23)	30(69.77)			10(23.26)	33(76.74)			35(76.74)	8(23.26)		
Lymph node metastasis													
Positive	31	5(16.13)	26(83.87)	1.653	0.198	4(12.90)	27(87.10)	0.652	0.419	13(41.94)	18(58.06)	11.611	≤0.001*∗*
Negative	45	13(28.89)	32(71.11)			9(20.00)	36(80.00)			36(80.00)	9(20.00)		

*∗* P<0.05, statistically significant.

**Table 3:** Associations of VM formation with EPO/EPO-R expression of CSCC.

*VM*	n	EPO				EPO-R			
		- (%)	+ (%)	*r*	*P*	- (%)	+ (%)	*r*	*P*
Positive	27	2(7.41)	25(92.59)	0.284	0.013*∗*	6(22.22)	21(77.78)	-0.101	0.386
Negative	49	16(32.65)	33(67.35)			7(14.29)	42(85.71)		

*∗* P<0.05, statistically significant.

## Data Availability

The data used to support the findings of this study are available from the corresponding author upon appropriate request.
